# Hippocampal Mechanisms Underlying Impairment in Spatial Learning Long After Establishment of Noise-Induced Hearing Loss in CBA Mice

**DOI:** 10.3389/fnsys.2018.00035

**Published:** 2018-07-24

**Authors:** Lijie Liu, Chuanying Xuan, Pei Shen, Tingting He, Ying Chang, Lijuan Shi, Shan Tao, Zhiping Yu, Richard E. Brown, Jian Wang

**Affiliations:** ^1^Department of Physiology, Medical College, Southeast University, Nanjing, China; ^2^Institute of Life Sciences, Southeast University, Nanjing, China; ^3^School of Communication Science and Disorders, Dalhousie University, Halifax, NS, Canada; ^4^Department of Psychology and Neuroscience, Dalhousie University, Halifax, NS, Canada

**Keywords:** noise-induced hearing loss, learning/memory, Morris water maze, hippocampal gene expression, neurogenesis, adult mice

## Abstract

Sensorineural hearing loss (SNHL) has been demonstrated in many clinical reports as a risk factor that promotes the development of cognitive impairment. However, the underlying neurological mechanisms are not clear. Noise exposure is one of the most common causes of SNHL. Although noise exposure causes relatively less damage to general health as compared with other methods for creating hearing loss (such as ototoxicity), it does impair cognitive function. Many studies have shown that the noise-induced cognitive impairment occur via the oxidative stress induced by the noise. In those studies, the effects of the noise-induced hearing loss induced (NIHL) were not addressed. Previously, we have demonstrated in the CBA/CaJ mouse model that oxidative stress was transient after a brief noise exposure, but the NIHL was permanent. In addition, NIHL was followed by a declined cognitive function and decreased hippocampal neurogenesis that were developed long after the oxidative stress disappeared. Therefore, NIHL can cause cognitive impairment independent of its stress effect and can serve as a model to investigate the relationship between hearing loss and the development of cognitive impairment. In the present study, we further demonstrated that the oxidative stress produced by the brief noise exposure did not damage the stem cell bank of hippocampus that was evaluated shortly after the noise exposure. In addition to the reduction in the rate of cell proliferation in hippocampus that was found previously, we found that the NIHL significantly reduced the promoting effect of learning activity on various stages of hippocampal neurogenesis, accompanied by the reduction in learning-induced expression of immediate early genes (IEGs) in hippocampus. Since the MWM-tested spatial function does not directly require auditory input, the results provide evidence for the maintenance role of auditory input on the cognitive function; the reduction of IEG expression that is required in memory-formation may be the initial step in blocking the effect of learning activity on neurogenesis in subjects with NIHL.

## Introduction

Hearing loss (HL) has been recognized as a promoting factor for cognitive decline with aging (Lin and Albert, 2014; Lin et al., 2014; Hardy et al., 2016; Park et al., 2016). However, the underlying mechanisms remain to be discovered. Since noise-induced hearing loss (NIHL) is a major type of acquired sensorineural HL, which interacts with age-related HL (Nelson et al., 2005; Yang et al., 2015; Hederstierna and Rosenhall, 2016; Liberman, 2017), and is quantitatively controllable in a laboratory setting, it is ideal for the investigation how HL impacts cognitive decline. Noise exposure has long been recognized as a cause of cognitive deficits through its direct effect on oxidative stress during and shortly after the noise exposure. The noise effect on oxidative stress has been the focus of previous studies, but the effects of HL have been largely ignored (Cui et al., 2009; Cheng et al., 2011; Jauregui-Huerta et al., 2011; Wright et al., 2014).

In our previous study (Liu et al., 2016), a brief noise exposure was used to create a permanent HL in CBA/CAJ mice, but the oxidative stress was only temporary, lasting less than a week. Using this mouse model, we demonstrated that NIHL, independent of the direct effect of noise on oxidative stress, impaired cognitive function in mice that were tested long after the establishment of NIHL. Furthermore, we found that the cognitive decline was associated with a reduced rate of cell proliferation in the dentate gyrus (DG) of the hippocampus.

Since the hippocampus is an important brain region for learning and memory, the present study further investigated the hippocampal mechanisms of NIHL on cognitive decline using the CBA/CAJ mouse model of NIHL. We first ruled out the possibility that the reduced cell proliferation in the hippocampus long after NIHL establishment is the result of the early noise-induced damage to the stem cell bank of the hippocampus via oxidative stress. In agreement with previous studies showing that neurogenesis was enhanced by learning in general (Gould et al., 1999) and specifically by spatial learning in the Morris water maze (MWM) (Ambrogini et al., 2000; Epp et al., 2007, 2010; Sisti et al., 2007; He et al., 2009; Zeng et al., 2016), we demonstrated a rescue effect of MWM training on the survival of newly generated hippocampal cells and proved that this rescue effect was much weaker in mice with NIHL. Consequently, neuronal differentiation and the integration of new neurons into the neural network were reduced in the mice with NIHL as compared with the control. Moreover, the MWM-induced immediate early gene (IEG) expression was lower in the mice with NIHL than that in the control mice.

## Materials and Methods

### Grouping and Experimental Outline

**FIGURE 1:**
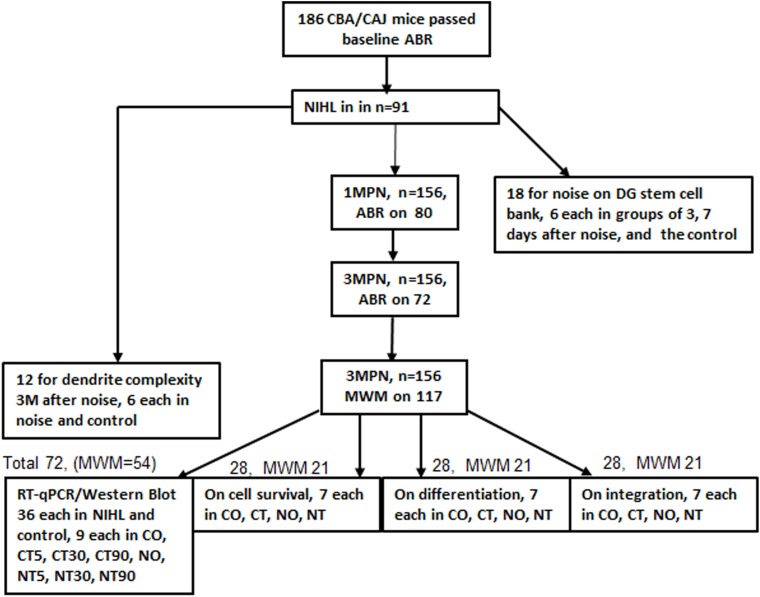
Summary of animal use and main procedures. ABR, auditory brainstem response; MWM, Morris Water Maze; 1/3MPN, 1 or 3 months post noise; CO, control (no noise), not trained with MWM; CT, control and trained with MWM; NO, NIHL, not trained; NT, NIHL and trained.

CBA/CAJ mice were chosen based upon their null connection with age-related HL (AHL); only male mice were used. They were obtained at 6–8 weeks of age from the Experimental Animal Center of Shanghai Super-B&K Laboratory Animal Corp. Ltd., Shanghai, China. The initial hearing status of these mice were measured with auditory brainstem response (ABR), and the 186 mice that passed the ABR test were used in the experiment. The detailed use of those mice is summarized in **Figure 1**. Approximately half of the animals were given noise exposure to create NIHL. Mice (18) were used to measure the immediate impact of noise on the bank of stem cells in the DG (12 in the noise group: six each at 3 and 7 days past noise, and six in the control group). Twelve mice were used to measure the impact of NIHL on dendrite complexity at 3 months post noise (3MPN, six each in the control and NIHL group). The remaining mice (*n* = 156) were used in the four experiments. ABR was administered at 1MPN in control and noise exposed mice and repeated at 3MPN on the same mice. MWM training was performed in the targeted mice at 3MPN after the final ABR. A total of 84 mice were used to measure the impact of NIHL and MWM training on neurogenesis in three experiments (28 in each): (1) the survival of newly proliferated cells, (2) neuronal differentiation, and (3) network formation of newly proliferated neurons. Each of the 28 mice were further divided into the four subgroups based upon the hearing (control/NIHL) and whether MWM training was given (CO, control, not trained; CT, control, trained; NO, NIHL, not trained; NT, NIHL, trained; with *n* = 7 in each). A total of 72 mice were used in the fourth experiment, measuring the impact of NIHL and MWM training on IEG expression in PCR and Western Blotting (WB; 36 control, 36 with NIHL). In each group, the mice were further divided into four subgroups (nine in each), one for mice receiving no MWM training (CO, control, not trained; N0, NIHL, not trained), and the others for mice from which brain samples were obtained at three time-points (5, 30, and 90 min) after MWM training (named as C5/N5, C30/N30, C90/N90). For various reasons, useful data were obtained from 171 mice. The sample size for the data analyses are specified in the results. Both the control and the NIHL groups were further divided into subgroups according to whether or not the MWM training was conducted.

All procedures were performed in accordance with the guidelines in the ethics protocol approved by the University Committee for Laboratory Animals of Southeast University, China (Permit number: SCXK2011-0003). During the experiment, the mice were housed in a Specific Pathogen Free (SPF) room with temperature controlled between 18 and 22°C, with 12/12 light/dark cycles, and air exchange rate of 10–20 times/h. Up to five mice were housed in one cage (∼60 l in volume), with free access to water and food. The ambient noise level was lower than 50 dB(A) SPL. All the tests were done during the light phase. They were fed with a standard diet of laboratory rodent chow for SPF mice (provided by Xietong Organism, Nanjing China).

### Hearing Evaluation With ABR

The hearing status was evaluated with ABR in each mouse when they were recruited. Otoscopic inspection was performed to rule out subjects with middle-ear infections. A mouse was rejected if it’s ABR thresholds were >10 dB higher than the mean at more than two adjacent frequencies tested or >15 dB higher than the mean at more than one frequency. The mice were anesthetized with pentobarbital (60 mg/kg, i.p.), the body temperature was maintained at 37.5–38°C with a thermostatic heating pad during the recording and the recovery period after the test. The generation of the auditory stimuli and the recording of the responses were done using hardware and the BioSigRZ software from Tucker–Davis Technology (TDT system III, Alachua, FL, United States). The acoustic system for stimulus delivering was calibrated by using a microphone system equipped with a 1/4 inch microphone (2520, Larson & Davis, Depew, NY, United States) and a microphone amplifier (12AK, G.R.A.S. Sound & Vibration, Twinsburg, OH, United States). Using the calibration module of the BioSigRZ software, the sound level was first tested at fixed output voltage of 1.414 V across 3000 points in ratio steps from 1 to 50 kHz. The difference of the detected sound level and targeted value (90 dB SPL) was recorded at each frequency and corrected by adjusting the output voltage. The stimuli were 10-ms tone bursts of different frequencies from 2 to 32 kHz in octave steps, with a rise-fall time of 1 ms. The acoustic signals were played through a broadband speaker (MF1 from TDT), which was placed 10 cm in front of the animal’s head. The tone bursts were presented at the rate of 21.1/s. The evoked responses were picked up by three subdermal electrodes, amplified 20 times, and digitized via a pre-amplifier (RA16PA) with a filter between 100 and 3000 Hz, and averaged up to 1000 times. The non-inverting electrode was inserted at the vertex of the head, and the reference and grounding electrodes were placed behind the two earlobes. At each frequency, the test was performed in a descending sequence from 90 dB SPL in 5-dB steps until the ABR response disappeared. The ABR waveforms were read buy two independent researchers. The ABR threshold was determined as the lowest sound level at which the tracked peak III was detectable and repeatable.

### Establishing NIHL

Noise exposure was given to awake mice, which were kept in a metal-wire cage, allowed to freely move with access to food and water. The two loudspeakers used for noise exposure (one was a low-frequency woofer and the other was a high-frequency tweeter) were positioned 60 cm above the cage floor. Electrical Gaussian noise was delivered to the speakers via power amplifier. The acoustic energy was mainly distributed flat between 1 and 20 kHz, as previously reported (Wang et al., 2011). The noise was given near the upper limit of the system: 123 dB SP for one session continuously for 2 h. The sound level was monitored during the exposure using a 1/4-inch microphone linked to a sound level meter (Larson Davis 824, Depew, NY, United States). ABR was repeated at 1MPN and 3MPN in a portion of mice in both control and NIHL groups.

### MWM Test

The MWM test was performed in a similar way as described previously (Bonaccorsi et al., 2013; Mendez-Couz et al., 2014; Liu et al., 2016). The grouping information was blinded from the tester who ran the MWM. The mice were moved to the room at least 1 h before the MWM was performed. The swimming pool was 122 cm in diameter. It was filled with tap water at 21–22°C. The water depth was 14 cm, 0.5 cm over the platform, which was 9 cm in diameter and made of transparent plastic. The pool was surrounded by blue curtain and four spatial cues made of black-and-white plastic boards of different shapes were placed in four quadrants around the pool, just above the water surface. The MWM procedure was modified from what was reported previously (Vorhees and Williams, 2006), which included 1-day of cued learning, followed by a 5-day spatial acquisition (or navigation) phase, and a 1-day probe test. Four trials were given to each mouse in each of the cued learning and spatial acquisition days, and three trials were given in the probe test day. Each trial was 60 s, with inter-trial intervals of 15 min. The starting quadrant was randomized across trials. The target quadrant of hidden platform was counterbalanced across groups. In the 1-day cued learning, a 12-cm high black-and-white flag was placed on the platform, and the spatial cues around pool were removed. The mice learnt to swim to the platform by orienting to the flag. In the spatial acquisition phase, a trial was considered successful when the mouse climbed onto the invisible platform within 60 s and stayed on it for more than 5 s. The escape latency was calculated between the time the mouse was placed into the water and the time it climbed on to the platform. If the platform was not found within 60 s, the mouse was guided to it. In the probe test, the platform was removed. Three trials were performed, each trial was also 60 s. The number of times the mouse swam across the location of the platform, the duration, and the swim distance of the mouse in the targeted quadrant were recorded as the index of spatial memory.

### Hippocampal Neurogenesis

The brain was harvested from mice that were deeply anesthetized with pentobarbital (100 mg/kg, i.p.) and fixed with open-chest cardiovascular perfusion of 4% paraformaldehyde in PBS buffer followed by post-fixation in the same fixative at 4°C for 24 h. The brain tissue block was then immersed in 30% sucrose, dehydrated at 4°C until it sunk to the bottom, embedded in optimum cutting temperature (OCT) compound and then frozen in a -80°C freezer. The frozen block was cut into a series of 25-μm thick slices using a microtome (Leica Cryostat Microtome 1900, Heidelberger, Germany). One slice from every 10 was chosen for data processing.

#### Noise Exposure on Stem Cell Bank

The impact of the noise exposure on the bank of hippocampal stem cells in DG region was observed at 3 and 7 days post noise exposure (3DPN and 7DPN) by counting the BrdU+ cells. BrdU was given at the dose of 50 mg/kg 4 h before the animals were sacrificed. The hippocampal slices were stained first with an antibody against BrdU {1:500, Anti-BrdU antibody [BU1/75(ICR1)], Rat monoclonal, Abcam, ab6326} at 4°C overnight, and followed by the treatment with the second antibody [1:1000, Donkey Anti Rat IgG H&L (Alexa Flour 488^®^), Abcam, ab150153] at 37°C for 1 h. The slice was then treated with a solution containing the first antibodies against Nestin and GFAP (1:100, Anti-Nestin, clone rat-401, Mouse monoclonal, Millipore, MAB353; 1:250, Anti-GFAP antibody, Rabbit polyclonal, Abcam, ab7260)at 4°C overnight, followed by a mixture of the second antibodies [1:1000, Cy 3-conjugated AffiniPure Goat Anti-Mouse IgG H&L, Jackson, 115-165-166; 1:500, Donkey anti-Rabbit IgG (H + L) secondary Antibody, Alexa Fluor^®^ 647 conjugate, Life Invitrogen, A-31573]. The triple positive cells (BrdU+/Nestin+/GFAP+) were counted as the stem cells.

### NIHL Impact on the Promoting Effect of MWM on Neurogenesis

The impact of NIHL on the ability of MWM learning-and-memory training to facilitate hippocampal neurogenesis in DG was observed at 3MPN in terms of (1) the survival of the newly proliferated cells (rescue effect), (2) neuronal differentiation of newly proliferated cells, and (3) the integration of neurons into a neural network. To observe the rescue effect of MWM training on cell survival after proliferation, BrdU was injected (200 mg/kg, Bid i.p.) 1 week before MWM training. The brain was harvested 24 h after the final day of MWM training (2 weeks after BrdU injection), and the hippocampus was sliced to count the BrdU+ cells in the DG region. This was done in the same way as it was for the observation of stem cells. To observe the effect of MWM on the neuronal differentiation of the newly generated DG cells, the mice were injected with EdU (100 mg/kg, Bid, i.p.) 1-week before the MWM and sacrificed 2 weeks after the MWM. The coronal sections of the whole brain were obtained in the thickness of 25 μm from between 0.94 and 4.04 mm posterior to the bregma (Paxinos and Franklin, 2001). One slice in every 10 was selected for the observations listed above. The hippocampal slices were treated with double staining against EdU and NeuN. The EdU signal was detected with a commercial kit (Click-iT^®^ EdU Imaging Kit with Alexa Fluor^®^ 488 Azides, Life Invitrogen, C10337) in accordance with the manufacturers’ instructions. After EdU staining, the slices were further stained with the first antibody against NeuN (1:500, Anti-NeuN, clone A60, mouse monoclonal, Millipore, MAB377, 4°C overnight) and then the second antibody [1:1000, Alexa Flour^®^ 568 goat anti-mouse IgG1(y1), Invitrogen, A21124, 37°C for 1 h]. To observe the effect of MWM training on neural integration, the mice were sacrificed 90 min after the MWM probe test, and the hippocampal slices were stained with the first antibody against Arc (Arc-C-7: sc-17839, Mouse monoclonal, Santa Cruz, United States, 4°C overnight) and the second antibody (Cy^TM^ 3-conjugated AffiniPure Goat Anti-Mouse IgG H&L, Jackson, 115-165-166, United States, 37°C for 1 h).

### Effect of NIHL on Dendrite Tree Complexity

The impact of NIHL on neural fiber branching (complexity) was observed at 3MPN in mice without MWM training. The brains were harvested from mice under deep anesthesia and the hippocampi were dissected out and immersed into the mixture of the A and B solution of the reaction kit (FD Rapid GolgiStain^TM^ Kit, FD NeuroTechnologies, Inc., United States) for 15 days with one change of the solution every 24 h. The hippocampal tissues were then embedded with TFM material (Cat# TFM-C, TBS, United States), and frozen at -80°C for slicing at 100 μm thickness. The slices were dehydrated with graded alcohol (from 50% to 100%, 2 min each) and made transparent with Dimethylbenzene for 5 min. Images of single neurons were taken under a microscope (Olympus BX53, Japan). Sholl analysis was performed on the isolated neuron images using the ImageJ software plugin named NeuronJ. Using this plugin, a series of concentric circles of different radii, centered at the cell body, were created. The complexity of the dendrite trees was measured by the number of intersected dendrites with each circle and the total length of all the branches of a single neuron.

### PCR and WB

The hippocampi were quickly harvested from the mice that were decapitated under deep anesthesia with pentobarbital (100 mg/kg, i.p.) and immediately frozen with dry ice and stored in -80°C freezer. RNA and protein were extracted using the cold TRIzol reagent (Life Invitrogen, Cat. No. 15596-018, United States). The RNA was converted into cDNA using Prime Script^TM^ RT reagent Kit with gDNA Eraser: Takara, Japan (Applied Biosystems, United States). The level of targeted RNAs was tested in RT-qPCR using the StepOnePlus^TM^ Real-Time PCR System (Thermo Fisher Scientific). The PCR reactions were performed in a total volume of 20 μL containing 10 μL of SYBR Premix Ex Taq^TM^: Takara, Japan (Applied Biosystems, United States) and 0.4 μl of ROX Reference Dye (50×). The PCR procedures included denaturation at 95°C for 30 s, followed by 40 cycles of amplification/denaturing at 95°C for 5 s and annealing for 30 s at 60°C. Quantification of expression from the cycle of threshold (Ct) was calculated using Step One Software v2.3 (Applied Biosystems) and the 2^-^∆ ∆ ^CT^ method (Livak and Schmittgen, 2001). Three genes (GAPDH, β-Actin, and Pgk1) were chosen as candidates for endogenous reference genes. The stability of these genes was evaluated using geNorm, NormFinder, and BestKeeper software. The outcome revealed that the RNA level of GAPDH was stable across the samples of the different groups in the present study and therefore was used as the reference gene. The sequences of forward and reverse primers (**Table 1**) were designed by Shanghai Generay Biotech Co., Ltd.

**Table 1:** RT-qPCR primer and probe sequences used for amplification of cDNA.

Gene	Forward primer sequence	Reverse primer sequence
GAPDH	5′-AGAAGGTGGTGAAGCAGGCATC-3′	5′-CGAAGGTGGAAGAGTGGGAGTTG-3′
β-Actin	5′-TCGTGATGGACTCCGGTGAC-3′	5′-TCGTGGATGCCACAGGACTC-3′
Pgk1	5′-GTCGTGATGAGGGTGGACTT-3′	5′-TTTGATGCTTGGAACAGCAG-3′
CREB	5′-GCTTATTCTGGTGAGTACTAAGTCTTAATGAGT-3′	5′-GACAAAAGTCTTTATTTATTAACACCTTATTG-3′
Arc	5′-CTCAGAGGAGTTCTTAGCCTGTTCG-3′	5′-ATCTCAGCTCGGCACTTACCAAT-3′
BDNF	5′-TGAGCGTGTGTGACAGTATTAGCG-3′	5′-CATGGGATTACACTTGGTCTCGTAG-3′
Egr-1	5′-GTTCCCATGATCCCTGACTATCTG-3′	5′-GACTGAGTGGCGAAGGCTTTAATA-3′
c-fos	5′-TTTCAACGCGGACTACGAGG-3′	5′-GCGCAAAAGTCCTGTGTGTT-3′
Npas4	5′-AGCAAGAGCCTGAGCGAAAAGA-3′	5′-CTTGGTGGATCGGTACATGACTG-3′

The protein concentrations were measured by using a BCA protein assay kit (Beyotime Biotechnology, Shanghai, China). To separate proteins, the protein extracts (30 μg from each preparation) were added to a sodium dodecyl sulfate–polyacrylamide gel electrophoresis (SDS-PAGE) of different concentrations (8, 10, or 12% according to the molecular weights of the target proteins). After electrophoresis (60 V for 30 min and then 90 V for 90 min), the separated proteins were electrotransferred onto polyvinylidene fluoride (PVDF) membranes (Millipore, Bedford, MA, United States). After blocking with 5% non-fat milk, the membranes were incubated with primary antibodies against the reference proteins overnight at 4°C. The primary antibodies used were: anti-NPAS4 (goat polyclonal, ab109984, Abcam, United Kingdom), anti-Egr-1 (rabbit monoclonal, ab133695, Abcam, United Kingdom), anti-c-fos (rabbit monoclonal, #2250S, CST, United States), anti-Arc (mouse monoclonal, sc-17839, SANTA CRUZ, United States), anti-CREB (rabbit monoclonal, #9197, CST, United States), anti-Phospho-CREB (rabbit monoclonal, #9197, CST, United States), anti-BDNF (rabbit monoclonal, ab108319, Abcam, United Kingdom), anti-GAPDH (rabbit monoclonal, #5174, CST, United States). After incubation with primary antibodies, the membranes were washed with TBST four times, 8 min each and then incubated with horseradish peroxidase–conjugated secondary antibodies. The protein bands were visualized using an ECL Kit (catalog number WBKLS0100; Millipore, Billerica, MA, United States). Finally, a densitometry analysis was performed using ImageJ software to quantify the targeted protein using GAPDH as the reference.

### Statistics

All statistical analyses were performed using SigmaPlot 12.2 software. The ANOVA tests were performed before the *post hoc* tests. Non-parametrical comparison was performed if the data failed to pass normality tests. All data are presented as the mean ± SEM, unless otherwise specified. Significance was accepted at the level of *p* < 0.05.

## Results

### Hearing Loss After Noise Exposure

**FIGURE 2:**
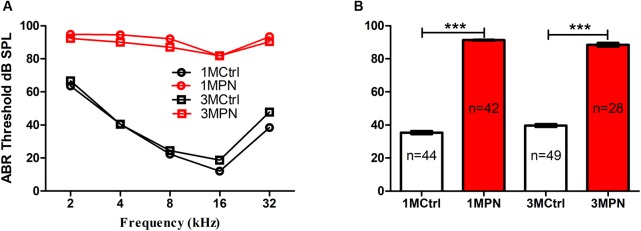
ABR frequency thresholds tested 1 and 3 months post noise exposure (1/3MPN) in noise groups and in age-matched control groups. **(A)** frequency-threshold curves. **(B)** frequency-averaged thresholds. ^∗∗∗^*p* < 0.001. Error bars: SEM.

In the present study, the hearing sensitivity was evaluated by ABR on every mouse at recruitment (before noise exposure). A portion of mice was re-tested at 1 and 3 months post the noise exposure (1MPN and 3MPN) in the mice with NIHL and in the age-matched control mice. The ABR frequency-threshold curves at the two timepoints largely overlapped within the control and the NIHL groups, but the thresholds in the mice with NIHL were much higher than those of the control mice (**Figure 2A**). **Figure 1B** compared the frequency-averaged thresholds between the groups at two timepoints. A two-way ANOVA demonstrated a significant effect for noise exposure (*F*_1,159_ = 3797.998, *p* < 0.001), but not for the timing factor. Pairwise *post hoc* tests (Tukey Method) showed that the thresholds of the noise group at the two timepoints were significantly higher than the control groups (for 1MPN, *q* = 69.117, *p* < 0.001; for 3MPN: *q* = 54.937, *p* < 0.001). There was no significant difference between the two timepoints within the control group and the noise group.

### NIHL Impaired Spatial Learning/Memory

**FIGURE 3:**
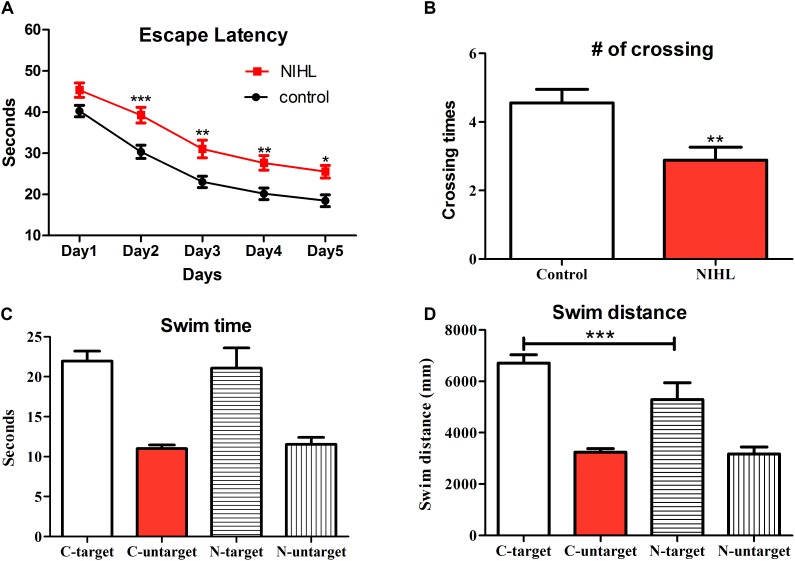
Deficits in spatial learning and memory 3 months after the NIHL. **(A)** Mean (±SEM) escape latency on each day of the MWM acquisition phase. **(B)** Mean (±SEM) number of times that mice swam across the location of the removed platform in the probe test. **(C,D)** Mean (±SEM) total time and swim distance in the targeted quadrant and the average of those measures over the three untargeted quadrants in the probe test. C, control; N, NIHL. ^∗∗∗^*p* < 0.001, ^∗∗^*p* < 0.01, and ^∗^*p* < 0.05. Sample size: *n* = 32 in the control group and *n* = 26 in the NIHL group.

Spatial learning ability and memory were tested in the MWM and compared between groups (*n* = 32 in the control and 26 in the NIHL group) on (1) the change in escape latency during the 5 days of the acquisition phase (**Figure 3A**), (2) the number of times mice crossed the location of the platform in the probe test in which the hidden platform was removed (**Figure 3B**), (3) the total time that mice stayed in the targeted quadrant and the average of the time that mice stayed in the three untargeted quadrants (**Figure 3C**) during the first probe test, and (4) the swim distance that of mice in the targeted quadrant and the averaged distance in the three untargeted quadrants. The MWM included four trials each day in the acquisition phase and three trials on the day of probe test; each trial lasted 60 s. A two-way ANOVA revealed a significant group (noise exposure) effect on escape latency (*F*_1,280_ = 50.57, *p* < 0.0001). *Post hoc* pairwise comparisons (Bonferroni method) showed that the escape latency of the noise-exposed group was significantly longer than that of the control group on days 2, 3, 4, and 5 of training (**Figure 3A**). In the first trial of the probe test, the control mice swam cross the location of the platform significantly more often than the NIHL mice (**Figure 3B**, *t*-test, *t* = 3.006, *df* = 58, *p* < 0.01). The swim distance in the targeted quadrant was found to be significant longer in the control [*Post hoc* test (Tukey method) after one-way mixed ANOVA, *q* = 3.939, *p* < 0.05]. However, there was no statistical difference in the total time the mice stayed in the target quadrant (**Figure 3C**).

### NIHL Impacts on Neurogenesis

#### The Noise Exposure Did Not Impact the Hippocampal Stem-Cell Bank

**FIGURE 4:**
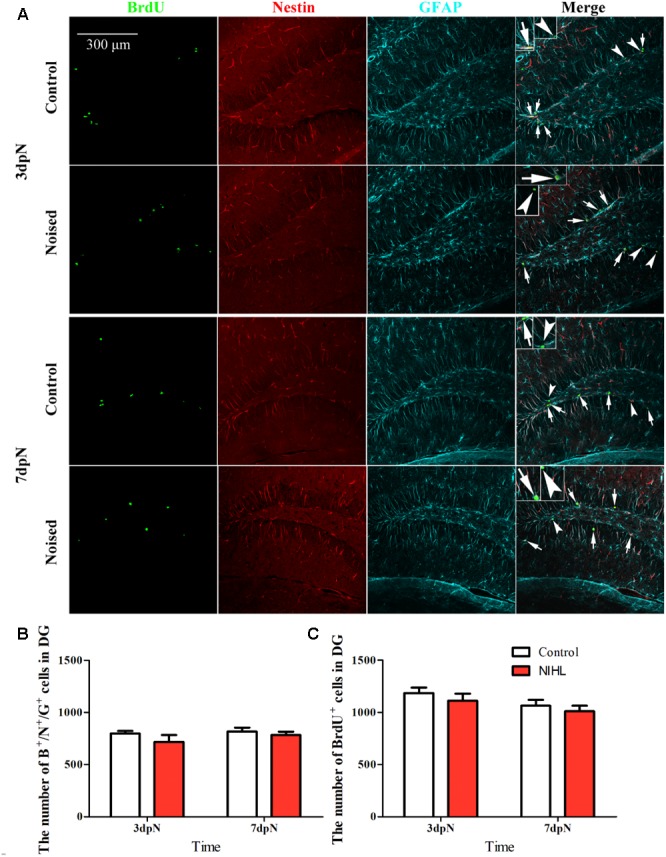
The impact of noise on hippocampal stem cell counts. **(A)** representative images of staining. The long arrows point to the triple-positive cells and the short arrows to the cells that were BrdU-positive only. Mean (±SEM) counts of **(B)** triple positive cells and **(C)** BrdU+ cells in the dentate gyrus (DG).

To rule out the possibility that the reduction in neurogenesis, long after establishment of NIHL, resulted from the immediate impact of noise-induced stress to the hippocampal stem cell bank, we compared the counts of newly proliferated cells (labeled by 5-bromo-2′-deoxyuridine, BrdU) and the stem cells (triple labeled by BrdU, Nestin, and GFAP) in the DG region between the control and the noise-exposed subjects at 3 and 7 days after the noise exposure. As shown in **Figure 4**, two-way between-group ANOVAs revealed that there was no significant effects of noise or timing on either triple-positive (Noise: *F*_1,21_= 1.958, *p* = 0.18, Timing: *F*_1,21_ = 1.053, *p* = 0.317) (**Figure 4B**) or BrdU-positive cell counts (Noise: *F*_1,21_= 1.163, *p* = 0.29; Timing: *F*_1,21_= 3.491, *p* = 0.08) (**Figure 4C**), nor was there a significant interaction (*F*_1,21_ = 3.497, *p* = 0.075).

#### NIHL Reduced the Rescue Effect of MWM on Cell Survival

**FIGURE 5:**
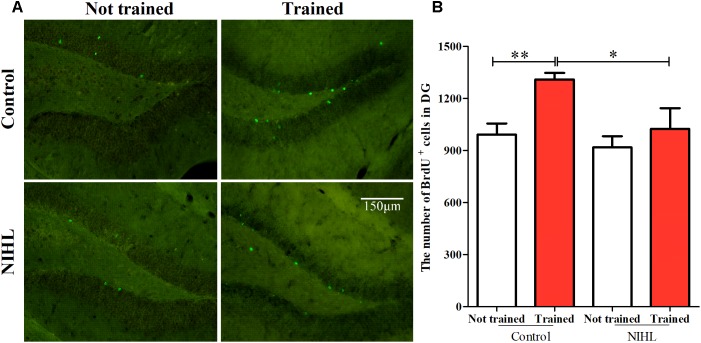
NIHL impact on proliferated cell survival in the DG of hippocampus. **(A)** representative images of BrdU staining of the 4 subgroups. **(B)** Mean (±SEM) BrdU+ cell counts in mice with NIHL and control mice, which had been trained with MWM or not trained. ^∗∗^*p* < 0.01, #*p* < 0.05. *n* = 6 or 7 in each subgroup.

Previously, we demonstrated that NIHL impacted neurogenesis by reducing the number of newly proliferated cells marked by DCX or Ki67 (Liu et al., 2016). In the present study, we examined whether NIHL impacted the rescue effect of MWM training on the survival of the newly proliferated cells. Cell survival was observed by *in vivo* BrdU labeling and the count of the BrdU+ cells 2 weeks after the labeling. In both the control and the NIHL groups, the mice were divided into two subgroups (*n* = 6 or 7 in each), one subgroup was given MWM training 1 week after the BrdU injection (trained), while the other was given no MWM training (not-trained). This 2 × 2 grouping allowed us to observe the impact of NIHL on the passive survival of the newly proliferated cells, the rescue effect of MWM, and the impact of NIHL on the rescue effect (**Figure 5**). Statistically significant effects of NIHL (*F*_1,22_ = 5.074, *p* < 0.05) and MWM training (*F*_1,22_ = 7.075, *p* < 0.05) were demonstrated by a two-way ANOVA. However, no significant interaction between the two factors was seen (*F*_1,22_= 1.721, *p* = 0.203). The number of BrdU+ cells in the trained control mice were significantly higher than in the not-trained controls (*post hoc* Tukey test, *q* = 3.972, *p* < 0.01), but this effect was not significant in the NIHL group (**Figure 5B**). Consequently, the BrdU+ cell counts in the trained mice with NIHL were significantly lower than in the trained control mice (*q* = 3.710, *p* < 0.05).

#### NIHL Reduced Promoting Effect of MWM on Neuronal Differentiation in Hippocampus

**FIGURE 6:**
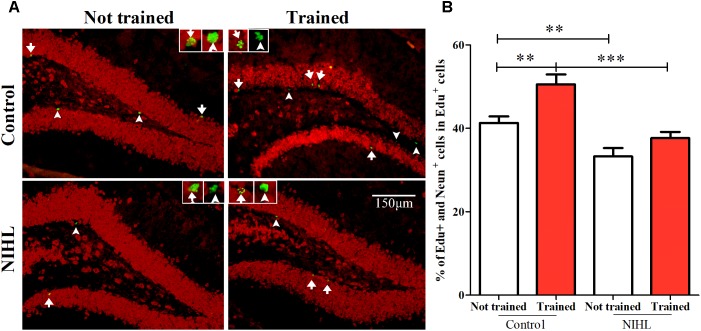
Impact of NIHL on neuronal differentiation in hippocampus. **(A)** representative images of EdU/NeuN double staining from the 4 subgroups. **(B)** Mean (±SEM) percentage of cells that were double labeled in the pool of cells labeled with EdU. ^∗∗^*p* < 0.01, ^∗∗∗^*p* < 0.001.

The newly proliferated hippocampal cells were labeled by *in vivo* injection of 5-ethynyl-2′-deoxyuridine (EdU), and those which differentiated to neurons were identified by double labeling with NeuN (**Figure 6A**). A 2 × 2 design (*n* = 5 or 6 in each subgroup) and a similar time line as that used in observing the rescue effect of MWM were utilized. The averaged percentage of EdU+/NeuN+ cells in EdU+ cell pools was increased by MWM training in the control mice, but not in the mice with NIHL (**Figure 6B**). A two-way ANOVA showed significant effects of NIHL (*F*_1,17_ = 31.014, *p* < 0.001) and MWM training (*F*_1,17_ = 13.373, *p* < 0.01) on neuronal differentiation, but no significant interaction between the two factors (*F*_1_ = 1.667, *p* = 0.214). Overall, fewer proliferated cells became neurons in the NIHL group than in the control group (**Figure 6B**), and the effect of MWM training was only significant in the control group (Tukey test, difference of mean = 7.267%, *q* = 5.172, *p* = 0.002). There was a significantly higher percentage of neurons in the control-trained mice than in the NIHL-trained mice (*q* = 7.014, *p* < 0.001, **Figure 6B**). The percentage of neurons in the control-not trained mice was also significantly higher than that in the NIHL-not trained mice (**Figure 6B**, *q* = 4.188, *p* = 0.009).

#### NIHL Reduced the Promoting Effect of MWM on Neural-Network Integration

**FIGURE 7:**
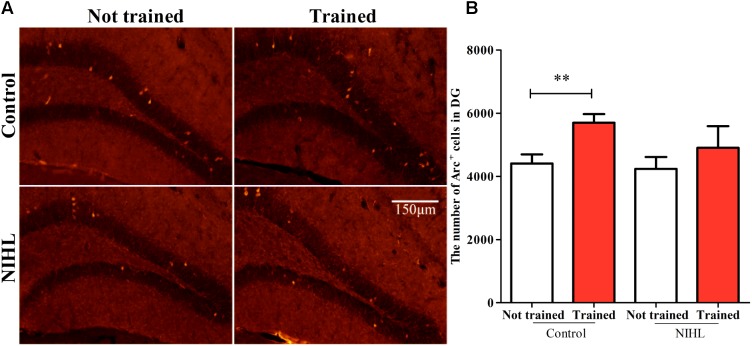
Impact of NIHL on training-promoted integration of new neurons into neuronal networks in the hippocampus. **(A)** representative image of Arc stained DG sections. **(B)** Mean ± SEM number of Arc+ cell counts in the control and NIHL mice given MWM training or not trained (*n* = 6 in each subgroup). ^∗∗^*p* < 0.01.

The number of neurons integrated into the neuronal network from the newly differentiated neurons in the DG zone of hippocampus was identified by Arc staining and was compared across the four subgroups (*n* = 6 in each, **Figure 7A**). A two-way ANOVA showed no significant effect of NIHL (*F*_1,21_= 1.55, *p* = 0.226) but a significant effect of MWM training (*F*_1,21_ = 5.498, *p* < 0.05) on the number of Arc+ cells (**Figure 7B**). *Post hoc* Tukey test showed only a significant effect of training in the control group (*q* = 3.155, *p* < 0.05). Again, no significant interaction was found between NIHL and MWM training (*F*_1,21_ = 0.440, *p* = 0.514).

#### NIHL-Reduced Neural Fiber Complexity

**FIGURE 8:**
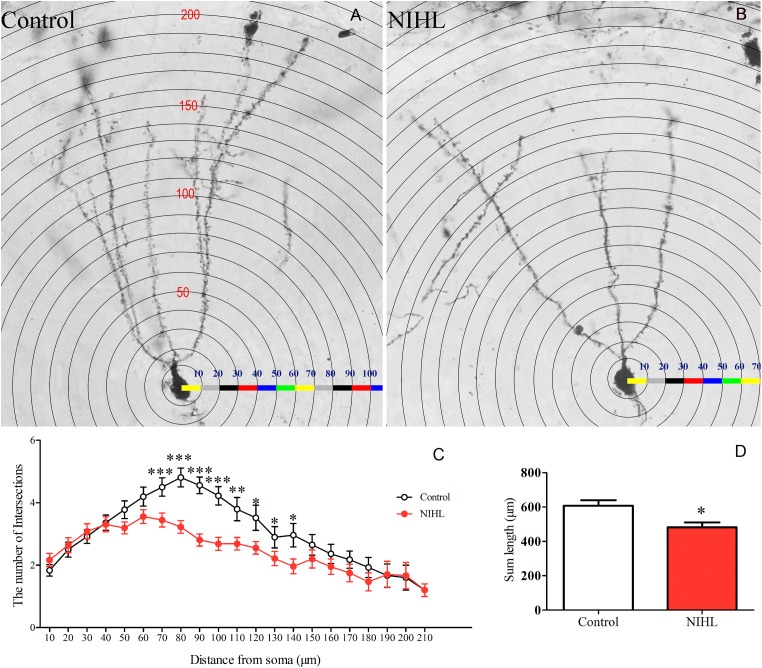
Impact of NIHL on dendrite complexity. Representative images of Golgi staining of neurons from **(A)** control and **(B)** NIHL mice. **(C)** The number of branches crossing the concentric circles at different distances from the body of the neuron. **(D)** Mean (±SEM) total length of nerve fibers of each neuron in control and NIHL mice. ^∗^*p* < 0.05, ^∗∗^*p* < 0.01, ^∗∗∗^*p* < 0.001.

The dendrite trees of DG neurons were identified with Golgi staining and compared between the control and the NIHL groups (*n* = 6) at 3MPN (**Figures 8A,B**). The complexity of the dendrite trees was quantified with the Sholl analysis plugin in the ImageJ software. A total of 72 isolated neurons were analyzed, 36 in each group. The number of dendrite intersections with the concentric circles was measured as the number of dendrite branches as a function of the distance from the cell body was analyzed (**Figure 8C**). A two-way ANOVA showed a significant effect of NIHL (*F*_1,8_ = 30.016, *p* < 0.001), demonstrating that there were fewer branches in the mice with NIHL than that in the control mice. *Post hoc* tests (Tukey method) showed that this difference was significant over the distance from soma between 70 and 140 μm (indicated by asterisks in **Figure 8C**). The total length of the neural projections of each neuron in the NIHL group was significantly shorter than that of the control mice (*t*-test, *t*_10_ = 2.805, *p* = 0.017, **Figure 7D**).

### NIHL Impact on Gene Expression

**FIGURE 9:**
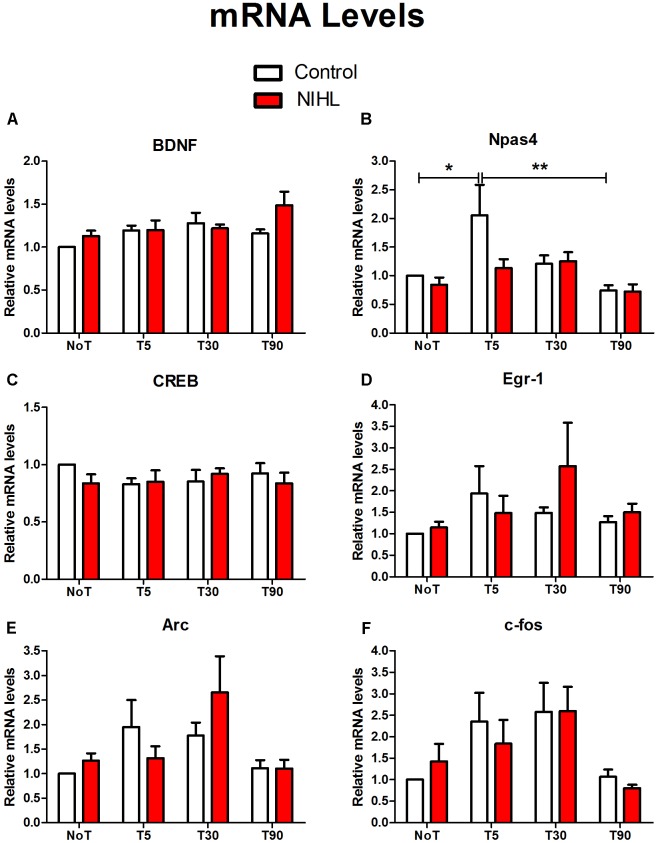
Impact of NIHL on IEG expression (mRNA) induced by MWM training. The samples from mice in the control and NIHL groups were sub-grouped as not-trained (NoT) and those from which the hippocampal DG samples were taken 5, 30, or 90 min after MWM training (T5, T30, and T90, respectively). The relative mRNA levels of BDNF **(A)**, Npas4 **(B)**, CREB **(C)**, Egr-1 **(D)**, Arc **(E)**, and c-fos **(F)** were compared with the mice that were not trained. A transient increase in Npas4 was induced by MWM training in the control mice, but not in the NIHL mice **(B)**. The sample sizes in each subgroup varied from 7–10. ^∗^*p* < 0.05, ^∗∗∗^*p* < 0.001.

**FIGURE 10:**
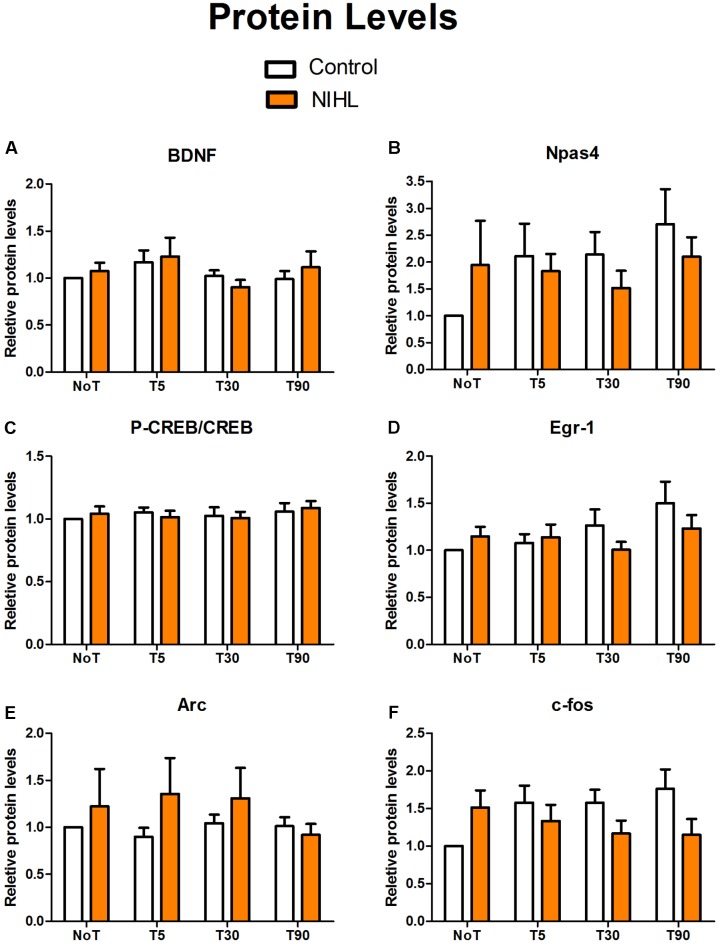
Impact of NIHL and MWM training on IEG protein levels. The hippocampal DG samples in the control and NIHL group were taken from mice that were not-trained (NoT) and 5, 30, or 90 min after MWM training (T5, T30, and T90, respectively). The relative protein levels of BDNF **(A)**, Npas4 **(B)**, p-CREB/CREB **(C)**, Egr-1 **(D)**, Arc **(E)**, and c-fos **(F)** were compared with the mice that were not trained. The sample sizes in each subgroup varied from 7 to 10.

The impact of NIHL on MWM training-induced changes in IEG expressions was observed at 3MPN using real-time quantitative polymerase chain reaction (RT-qPCR) for mRNA (**Figure 9**) and WB for proteins (**Figure 10**). The targeted genes included brain-derived neurotrophic factor (BDNF), cAMP-response element binding protein (CREB), neuronal PAS domain protein 4 (Npas4), Early growth response protein 1 (Egr-1 or zif268), activity-regulated cytoskeleton-associated protein (Arc), and c-fos. To examine the effect of MWM training, four subgroups were set up in each of the noise and control groups: one subgroup of not trained mice (NoT), and three subgroups of trained mice from, which hippocampal DG samples were obtained 5, 30, and 90 min after the final trial of the probe test in the last day of MWM (named as T5, T30, and T90, respectively).

While increased mRNA levels were seen in many genes after MWM training, the only significant change was demonstrated in Npas4. The two-way ANOVA for this gene demonstrated a significant effect in the time of sampling after MWM training (*F*_3,58_= 4.273, *p* = 0.009), but no significant effect of NIHL (*F*_1,58_= 2.059, *p* = 0.157). The Tukey *post hoc* pair-wise tests showed a significant temporal increase of Npas4 mRNA in the control group 5 min after the MWM training (*q* = 4.249, *p* = 0.02). The elevated Npase4 mRNA level was significantly decreased at 90 min (*q* = 7.755, *p* < 0.001). However, no such temporal increase was seen in the NIHL group, and no significant differences were found between the NIHL and the control groups at the time point of 5 min. For the other genes observed, there were no significant effects of MWM training or NIHL, as demonstrated in two-way ANOVAs (**Figure 9**).

**Figure 10** shows the relative levels of IEG proteins across samples. Two-way ANOVAs showed no significant effects of NIHL or MWM training on any of the IEG proteins, although Npas4 protein level appeared to be increased after MWM training in the control group as compared with the not-trained mice.

## Discussion

In the present study, young adult CBA/CAJ mice were briefly exposed to intense noise to produce NIHL, which was verified to be of a moderate-severe degree by ABR (**Figure 2**). Because the highest sound level in the ABR test was 90 dB SPL, the threshold was assigned as 95 dB SPL when no response was seen at 90 dB SPL. Mice with NIHL showed a poorer performance in spatial learning and memory in the MWM tested at 3MPN (**Figure 3**). The poorer performance in the MWM in the NIHL group was accompanied by the reduced effect of MWM training on hippocampal neurogenesis. This was demonstrated by the null effect of MWM in (1) the survival of newly proliferated cells (**Figure 5**), (2) neuronal differentiation after proliferation (**Figure 6**), and (3) integration of neurons into a neural network (**Figure 7**). The dendrite tree of hippocampal neurons had significantly fewer branches in the NIHL group (**Figure 8**). Furthermore, MWM training resulted in a temporal increase in Npas4 mRNA in the control group, but not in the NIHL group (**Figure 9**). However, the change of Npas4 protein was not significant (**Figures 9**, **10**).

### The Effect of Stress and HL

Similar to other stressors, noise-induced oxidative stress impairs hippocampal neurogenesis via increasing adrenal glucocorticoid (GC) hormones (Wang et al., 2013; Lucassen et al., 2014). However, the impact of acute stresses, such as those induced by our noise exposure, is likely to be transient (Lucassen et al., 2006, 2015; Hunter et al., 2015). In fact, the GC response to stress is under restrictive regulation and newly generated neurons in the hippocampus can shut off the stress induced GC responses (Opendak and Gould, 2011; Snyder et al., 2011). This is also the case following oxidative stress induced by brief noise exposure (Samson et al., 2008; Hayes et al., 2011). In our previous study, we also showed that the stress GC hormones and other stress indexing agents were upregulated only temporarily by a noise exposure similar to what was used in the present study (Liu et al., 2016). Therefore, the transient increase in GC hormones is unlikely the reason for the decline in cognitive functions and hippocampal neurogenesis observed long after the noise exposure (Liu et al., 2016). In the present experiment, we observed that the brief noise exposure did not change the stem cell bank in the hippocampus, measured at 3 and 7 days after the noise exposure (**Figure 4**). This result further rules out the possibility that the reduced neurogenesis seen 3MPN is a result of the early impairment of noise on the hippocampal stem-cell bank.

### Effect of HL on Neurogenesis

Adult neurogenesis in the hippocampus is a complex dynamic process consisting of multiple stages that are genetically and morphologically identifiable: (1) proliferation and expansion of nestin-expressing neural stem cells (NSCs) and progenitor cells that might differentiate into neurons, migration, and differentiation of doublecortin (DCX)-expressing neuroblasts, (2) survival of newly generated cells, (3) maturation and integration of new-born neurons (the growth of axon and dendrites), (4) the synaptogenesis and the establishment of functional connections with functional circuits (Kempermann et al., 2004, 2015; Ming and Song, 2011; Goncalves et al., 2016).

It has long been established that MWM, a spatial learning task that depends on the hippocampus and requires a concerted effort, can enhance the adult hippocampal neurogenesis by rescuing newly generated cells from death and promote their long-term survival (Gould et al., 1999; Anderson et al., 2011; Epp et al., 2013). The response of neurogenesis to spatial training was first demonstrated in rats by Gould et al. (1999). In that study, male rats received BrdU injection and were trained in the MWM 1 week later. The 1-week delay allows for progenitor cell differentiation into neurons, but not to their full maturation. Most adult-born hippocampal neurons are dying during the first 1–2 weeks after they are born (Cameron et al., 1993). However, as Gould et al. (1999) demonstrated, the animals submitted to MWM training showed an increase in the number of BrdU-labeled cells, indicating that the spatial learning enhanced the survival of adult generated neurons. This result has been supported by a number of studies that also described the positive effect of spatial learning on the survival of immature cells (Epp et al., 2007, 2010, 2013; Anderson et al., 2011).

In both neonatal C57 mice (Tao et al., 2015) and adult CBA/CAJ mice (Liu et al., 2016), we observed that poor performance in the MWM was associated with the degree of HL and with the decrease in cell proliferation in the hippocampus. In the present study, we moved a big step forward by demonstrating the reduced promoting effect of MWM training on hippocampal neurogenesis in the NIHL group. Specifically, NIHL suppressed (1) the rescue effect of MWM training on the survival of newly generated cells (**Figure 5**), (2) the ability of MWM training to promote neuronal differentiation (**Figure 6**), and (3) integration of neurons into a neural network (**Figure 7**). Furthermore, the dendrite tree was much less complex in the hippocampus of the mice with NIHL (**Figures 8**, **9**). At this moment, it is not clear how HL produces such a great impact on hippocampal neurogenesis. Since the impairment is related to a training paradigm that does not rely upon auditory function, we propose that the auditory input may play a baseline maintenance role across all stages of hippocampal neurogenesis.

### Effect of HL on MWM-Induced IEG Expression

Hundreds of genes have been associated with learning and memory (Mayford and Kandel, 1999; Paratore et al., 2006; Lee, 2014; Tamvacakis et al., 2015). IEGs are genes that are activated transiently and rapidly in response to a wide variety of cellular stimuli. They represent a standing response mechanism that is activated at the transcription level in the first round of responses to external stimuli, before any new proteins are synthesized. Learning-induced expression of IEGs provides a link between behavioral experience and the molecular events required to encode memory (Azami et al., 2006; Okuno, 2011). Genetic perturbations of IEGs or transcription factors that control activity-regulated gene expression thus often leading to deficits in neuronal plasticity and memory (Etkin et al., 2006; Ploski et al., 2008; Savonenko et al., 2009; Maddox and Schafe, 2011).

Most IEGs can be induced by a wide range of stimuli and are involved in processes essential to normal cellular function and survival. This suggests that their function may not be specific to learning related neuronal activity. However, Npas4 is specifically responsive to learning activity (Lin et al., 2008; Ramamoorthi et al., 2011). In the present study, a significant increase in Npas4 expression was seen after MWM training in the control group, but not in the NIHL group. This difference suggests that NIHL may impact hippocampal function via depressing IEGs. Consistent with previous reports, the increase of Npas4 is transient and rapidly induced after MWM (**Figure 9**) (Lin et al., 2008; Ramamoorthi et al., 2011). Since Npas4 is likely the molecular link between the learning related neuronal activity and memory (Sun and Lin, 2016), the null effect of the MWM training on Npas4 in the NIHL group may indicate where this link is broken down by HL.

Npas4 has been identified as the first gene that is increased by learning-related activities and the increased expression of this gene leads the increase of other IEGs (Lin et al., 2008; Ramamoorthi et al., 2011; Rudenko et al., 2013; Klaric et al., 2014; Madabhushi et al., 2015; Sun and Lin, 2016). However, we did not see significant increases of other IEGs following Npas4 elevation. The effect of MWM on the expression of other IEGs (such as c-fos, Egr-1 and Arc) has been observed in several studies, most of which were in rats (Carter et al., 2015; Barry et al., 2016; Farina and Commins, 2016; Barry and Commins, 2017). The MWM protocol and results varied across the studies. In one study, c-fos, Arc and Egr-1 were examined during and at different times after 1-day MWM training that had four trials. A temporary increase in the expression of those genes were seen in both DG, and both CA1 and CA3 regions that were examined by immunohistochemistry (IHC) against naïve controls (Carter et al., 2015). However, no significant differences were seen in the mRNA levels of those genes examined in PCR between MWM trained subjects and the swim control. This suggested that the increase in the expression level was likely due to the non-specific effect of swimming, rather than spatial learning and memory (Carter et al., 2015). In another study, Egr-1 was found to be increased in the CA1 and 3 but not in the DG region 90 min after 5-days of MWM training (Farina and Commins, 2016). No effect of MWM training was seen on c-fos in this study. However, different results were reported in multiple studies that conducted similar MWM training (Barry et al., 2016; Barry and Commins, 2017). In these studies, IEG expression appears to vary across different brain regions, and even across different cell types. For example, in one study using C57 mice, MWM training increased Egr-1 in mature granule cells but decreased it in immature granule cells (Huckleberry et al., 2015). This differential effect may be the reason for the negative results in the present study on many IEGs that were examined with PCR and WB. This suggests that IHC observation (Barry et al., 2016; Farina and Commins, 2016; Barry and Commins, 2017) or in situ hybridization (Laeremans et al., 2015) may be more suitable for the evaluation of learning/training effects on IEG expression that may vary across different regions of hippocampus.

## Conclusion and Future Directions

The present study provides clear evidence that suggests the adverse effect of NIHL on the cognitive functions tested in spatial learning and memory are independent from the stress of noise. Since the cognitive function tested with MWM training does not require auditory input, we hypothesize that the auditory input plays a maintenance role for the hippocampal function in general and HL may have reduced the general “learning-like” activity in the cognitive brain. The HL appeared to impair hippocampal cell proliferation and then reduce the promoting effect of the learning activity on later stages of hippocampus neurogenesis. Further research is needed to verify the impact of NIHL on the expression of learning/memory related genes, by using different quantification methods and to observe the potential changes in other cognitive brain regions beyond the hippocampus. The results in the present study have moved us one step forward in understanding the mechanism how HL impairs cognition. However, more research using different methods is needed to uncover the mystery. Recently, the levels of p-tau and lipofuscin were found to be increased in the hippocampus of C57 mice with NIHL, in association with the permanent decline in recognition memory long after the establishment NIHL (Park et al., 2018). Research is also needed to explore the potential influence of NIHL on the function of cognitive neurons to provide insight into how HL impacts the cognitive functions that are not directly related to hearing.

## Author Contributions

LL, CX, and PS conducted the experiment and data analysis. LL, LS, RB, and CX conducted the data analysis and paper writing. JW conducted research design, data analysis, and paper writing. All authors reviewed the manuscript.

## Conflict of Interest Statement

The authors declare that the research was conducted in the absence of any commercial or financial relationships that could be construed as a potential conflict of interest.
